# Size and depth of residual tumor after neoadjuvant chemoradiotherapy in rectal cancer – implications for the development of new imaging modalities for response assessment

**DOI:** 10.3389/fonc.2023.1209732

**Published:** 2023-09-05

**Authors:** Stefan D. van der Stel, Jose G. van den Berg, Petur Snaebjornsson, Iris M. Seignette, Mark Witteveen, Brechtje A. Grotenhuis, Geerard L. Beets, Anouk L. Post, Theo J. M. Ruers

**Affiliations:** ^1^ Faculty Technische Natuurwetenschappen (TNW), Group Nanobiophysics, Twente University, Enschede, Netherlands; ^2^ Department of Surgery, Netherlands Cancer Institute, Amsterdam, Netherlands; ^3^ Department of Pathology, Netherlands Cancer Institute, Amsterdam, Netherlands; ^4^ Faculty of Medicine, University of Iceland, Reykjavik, Iceland; ^5^ GROW School for Oncology and Developmental Biology, University of Maastricht, Maastricht, Netherlands; ^6^ Department of Biomedical Engineering and Physics, Amsterdam Cardiovascular Sciences, Cancer Center Amsterdam, Amsterdam Universitair Medisch Centrum (UMC), University of Amsterdam, Amsterdam, Netherlands

**Keywords:** tumor response, regression, rectal cancer, fragmentation, neoadjuvant, optical imaging, watch-and-wait

## Abstract

With the shift towards organ preserving treatment strategies in rectal cancer it has become increasingly important to accurately discriminate between a complete and good clinical response after neoadjuvant chemoradiotherapy (CRT). Standard of care imaging techniques such as CT and MRI are well equipped for initial staging of rectal tumors, but discrimination between a good clinical and complete response remains difficult due to their limited ability to detect small residual vital tumor fragments. To identify new promising imaging techniques that could fill this gap, it is crucial to know the size and invasion depth of residual vital tumor tissue since this determines the requirements with regard to the resolution and imaging depth of potential new optical imaging techniques. We analyzed 198 pathology slides from 30 rectal cancer patients with a Mandard tumor regression grade 2 or 3 after CRT that underwent surgery. For each patient we determined response pattern, size of the largest vital tumor fragment or bulk and the shortest distance from the vital tumor to the luminal surface. The response pattern was shrinkage in 14 patients and fragmentation in 16 patients. For both groups combined, the largest vital tumor fragment per patient was smaller than 1mm for 38% of patients, below 0.2mm for 12% of patients and for one patient as small as 0.06mm. For 29% of patients the vital tumor remnant was present within the first 0.01mm from the luminal surface and for 87% within 0.5mm. Our results explain why it is difficult to differentiate between a good clinical and complete response in rectal cancer patients using endoscopy and MRI, since in many patients submillimeter tumor fragments remain below the luminal surface. To detect residual vital tumor tissue in all patients included in this study a technique with a spatial resolution of 0.06mm and an imaging depth of 8.9mm would have been required. Optical imaging techniques offer the possibility of detecting majority of these cases due to the potential of both high-resolution imaging and enhanced contrast between tissue types. These techniques could thus serve as a complimentary tool to conventional methods for rectal cancer response assessment.

## 1 Introduction

Over the last decade, rectal cancer treatment has shifted towards organ preserving treatment, having the foremost advantage of improving the patient’s quality of life (1, 2). The standard-of-care for intermediate risk and locally advanced rectal cancer is neoadjuvant chemoradiotherapy (CRT) to reduce the size or extent of the tumor, followed by a total mesorectal excision (TME) where the rectum is surgically removed together with surrounding tissue and draining lymph nodes. Moreover, novel advances in rectal cancer treatment indicate the promising role of CRT in patients with bulky and distal tumors of the rectum, providing insights for organ preserving treatment in these complex tumors (3, 4). Since a TME often results in loss of organ function and considerable side effects, there is an increasing interest in organ-sparing treatment to improve the quality of life of patients. Patients with a good clinical response (defined as a near-complete or major response after CRT, with the possibility of residual tumor (5)) could receive additional local tumor treatment (e.g. a local tumor excision or internal boost radiation). In addition to CRT, novel advances in rectal cancer treatment have shown promising results with immunotherapy, especially in patients with microsatellite instable (MSI)-high tumors (6, 7).

In patients with a complete response (without any residual tumor) on the other hand non-operative management can be considered and these patients can be monitored according to watch-and-wait (W&W). After CRT, 20% of patients have a pathological complete response and 42-60% of patients have a good clinical response (8, 9). For organ-sparing treatment to become even more successful, it is important that clinicians can accurately identify the optimal treatment for each patient based on the degree of tumor response to CRT. However, the current workflow for response assessment has difficulty discriminating between patients with a good clinical response and a clinical complete response (10).

Response assessment is currently performed based on a combination of endoscopy, magnetic resonance imaging (MRI) and digital rectal examination. Endoscopic biopsies are only rarely used for initial response assessment because of frequent false positive results. Based on this *clinical* response assessment patients undergo further treatment or are enrolled in W&W. After surgery the resected tissue is analyzed by a pathologist, resulting in a *pathological* response assessment, which remains the gold standard. In approximately 15% of patients that are considered clinical incomplete responders, no residual tumor tissue is present upon histopathological evaluation of the resected specimen (11). These patients undergo major surgery where organ-sparing treatment could have been possible. Additionally, approximately 25% of patients thought to have responded completely based on a clinical evaluation still harbor unrecognized residual tumor (12). Based on the clinical evaluation these patients can be enrolled in W&W, but they developed a local regrowth requiring additional surgery (13). Improving the accuracy of response assessment thus holds the promise of improving treatment decisions and outcome for rectal cancer patients.

The difficulty of MRI to accurately assess tumor response can be explained by the fact that CRT can result in small tumor fragments scattered throughout fibrotic tissue (14). Not only does MRI have difficulty in discriminating between fibrosis and tumor tissue (15, 16) but a major concern in response assessment is also missing small fragments of residual vital tumor tissue, leading to the cautious strategy to perform major surgery whenever residual tumor tissue is suspected (17). CRT treatment can result in response patterns of either shrinkage or fragmentation of rectal tumors. Tumor shrinkage is characterized by a decrease in concentric tumor size, while fragmentation is defined by destruction of the main tumor mass after treatment and formation of small groups of tumor cells embedded in fibrosis. Fragmentation is reported in 40-80% of patients with rectal cancer (18, 19) and increases the chance of radiological understaging because of the difficulty of detecting small tumor fragments (20). While, to the best of our knowledge, the size distribution of tumor fragments in rectal cancer have not been published, a recent study showed that tumor fragment size in esophageal adenocarcinoma can be as small as several micrometers (18). With these dimensions, MR imaging lack resolution and accuracy in visualizing residual tumor fragments. Thus, to improve response assessment and treatment after CRT, an imaging technique is required that can detect small vital tumor fragments within fibrotic tissue.

To identify promising new techniques to improve response assessment in rectal cancer, one requirement placed on such a technique is that it can distinguish between vital tumor tissue and fibrosis. A second requirement is that the resolution needs to be high enough so it can detect small residual tumor fragments. While techniques like MRI can image the full body, for most other imaging techniques there is a trade-off between the resolution and imaging depth. Therefore, a third requirement is that a new technique needs to be able to image deep enough below the luminal surface so that it can detect residual vital tumor tissue in deeper tissue layers. While general response patterns in rectal cancer have been described in literature, no quantitative description of the size and depth distributions of residual tumor fragments after CRT has been given. Nonetheless, these quantitative measures potentially play a pivotal role in explaining why so many tumors are misclassified by current imaging methods and consequently could provide the theoretical framework more optimal imaging methods in the future. The aim of this study is to provide a quantitative histopathological description of the size and depth distributions of residual tumor tissue after CRT treatment which can be used to select promising new imaging techniques for response assessment based on their resolution and imaging depth.

## 2 Patients, materials and methods

### 2.1 Test cohort

Histological slides of rectal resection specimens from 30 patients with rectal tumors that had been treated with CRT and underwent rectal surgery were retrieved from the pathology archive at the Netherlands Cancer Institute. The original pathology report contained information on tumor regression grade (TRG), scored according to Mandard (21). Since we aimed to improve discrimination between a good clinical and a complete response, only cases with a substantial pathological response after CRT treatment were included - scored as Mandard TRG 2 (rare residual tumor cells and clusters scattered through fibrosis) or TRG 3 (increase in the number of residual tumor cells when compared to Mandard TRG 2, while fibrosis still predominates when compared to Mandard TRG 4).

This retrospective medical data/biospecimen study was carried out pursuant to Dutch legislation and international standards. Clinical information such as demographics and tumor characteristics were collected from the medical records (**Table 1**). Archival H&E slides were scanned using a PANNORAMIC^®^ 1000 scanner from 3DHISTECH at a 40x magnification.

**Table 1:** General patient and tumor characteristics.

Total	30	
Gender		
Male	19
Female	11
Age, *median (IQR)*	57 (54 – 69)
Neoadjuvant treatment		
Long course chemoradiation	18
Short course RT and immunotherapy within a trial (22)	5
Short course RT followed by chemotherapy	4
Short course RT	3
Interval between neoadjuvant treatment and surgery (weeks), *median (IQR)*	13 (10 – 17)
Type of surgery		
Abdominoperineal resection (APR)	13
Low anterior resection (LAR)	12
Transanal minimally invasive local excision (TAMIS)	5
Tumor type		
Well/moderately differentiated adenocarcinoma (low grade)	27
Poorly differentiated adenocarcinoma (high grade)	2
Mixed Neuroendocrine Non-Neuroendocrine Neoplasm (miNEN)	1
Mandard tumor regression grade		
2	17
3	13
Tumor invasion in rectum		
Mucosa/submucosa (ypT1)	5	
Muscularis propria (ypT2)	16	
Pericolic/mesorectal tissue (ypT3)	8	
Other organs/structures (ypT4)	1	

### 2.2 Assessment of tumor response

Indica Labs’ HALO software (v3.4.2986.185) (23) was used to classify tissue, normal mucosa, and tumor areas on the scanned histopathology slides, and to subsequently measure size and volume of tumor cell clusters and distances between tumor cell clusters. The DenseNet AI v2 plugin classifier was trained with 3 complete annotated slides, where a certified pathologist (JGvdB) annotated the full regions of background, normal mucosa, tumor and all other tissues. The classifier was trained for a total of 26345 iterations with a Cross-Entropy of 0.1. After training, performance of the classifier was verified by JGvdB in random slides included in this study. In total, 198 slides were examined, consisting of all H&E tumor slides per case.

First, we determined for each patient whether the response pattern was of fragmentation or shrinkage type. Tumor fragmentation was defined as clusters of cells which do not form a bulk and have at least 3 mm distance between fragments (18). If the response pattern was fragmentation, we measured the width (the short axis) of the widest tumor fragment per patient to be able to determine the ability of different imaging techniques to detect small fragments based on their resolution. We chose the width since that defines the required resolution – a long and narrow fragment of e.g. 3 by 0.05 mm would not be detected by a technique with a resolution of 1 mm. We chose the widest fragment per patient since the detection of only that fragment could already be enough to determine an incomplete response. In patients where the response pattern was of shrinkage type we measured the largest width of vital tumor tissue. For all patients we determined the shortest distance from the luminal surface to the vital tumor, in order to determine the ability of different imaging techniques to detect this residual tumor tissue based on the imaging depth. Finally, per patient we measured the tumor volume (based on all slides of a single patient) and the tumor area (based on the single slide with largest tumor area of each patient).

### 2.3 Statistical analysis

Statistical analysis was performed using IBM SPSS statistics v27 (SPSS Inc., United States). Normal distribution was assessed with the Shapiro-Wilk test. Statistical analysis for normally distributed data was performed with an unpaired t-test, and for non-normally distributed data using a Mann-Whitney test. A p-value ≤0.05 was considered statistically significant.

## 3 Results

### 3.1 Histopathological evaluation of tumor response

**Figure 1:**
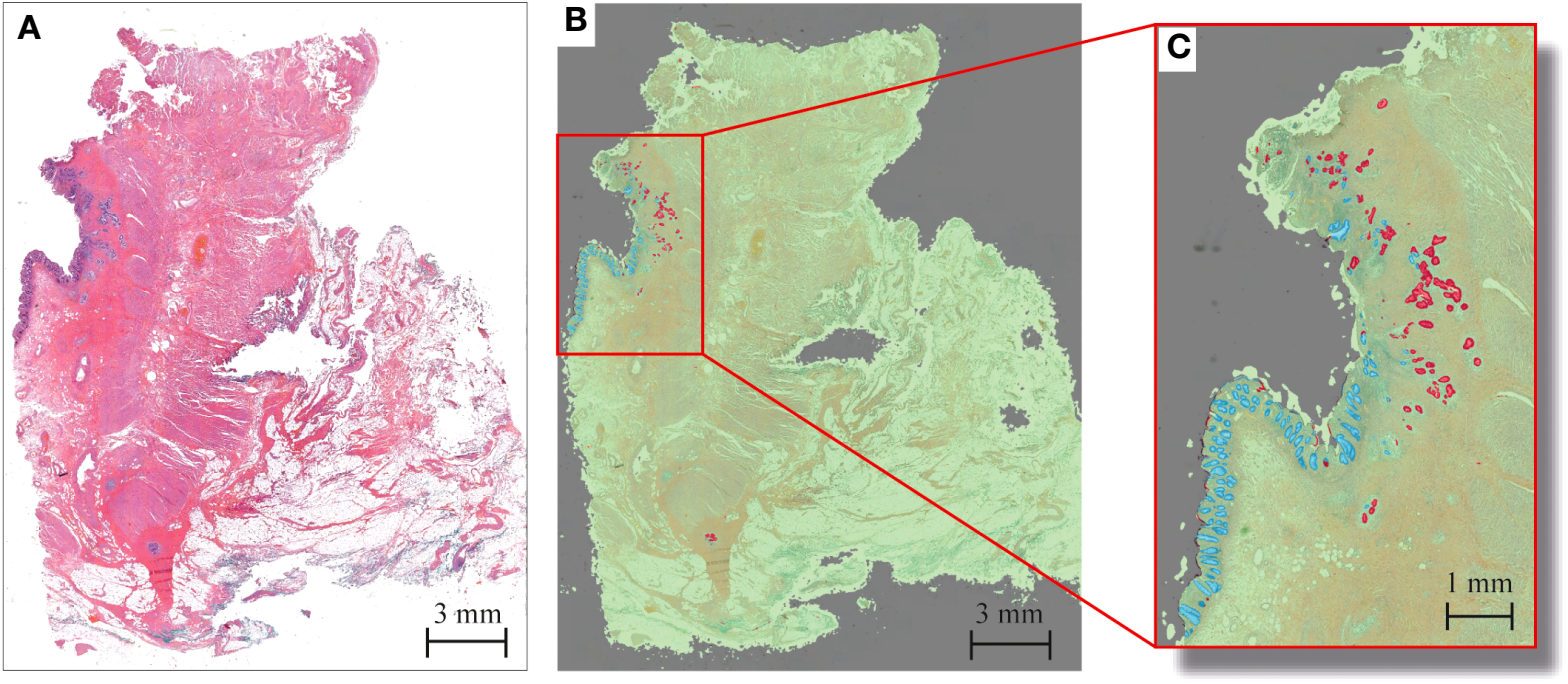
Example of tumor response segmentation by artificial intelligence HALO software in rectal cancer after CRT in a patient displaying Mandard TRG 2. **(A)** Haematoxylin and eosin (H&E) snapshot of tissue slide. **(B)** Corresponding tissue labels created by HALO tissue segmentation to enable easy tumor visualization. Red = tumor; blue = mucosa; green = other tissue; gray = background. **(C)** Zoom-in overview of tumor response pattern, with tumor fragments as small as several micrometers.

**Figure 2:**
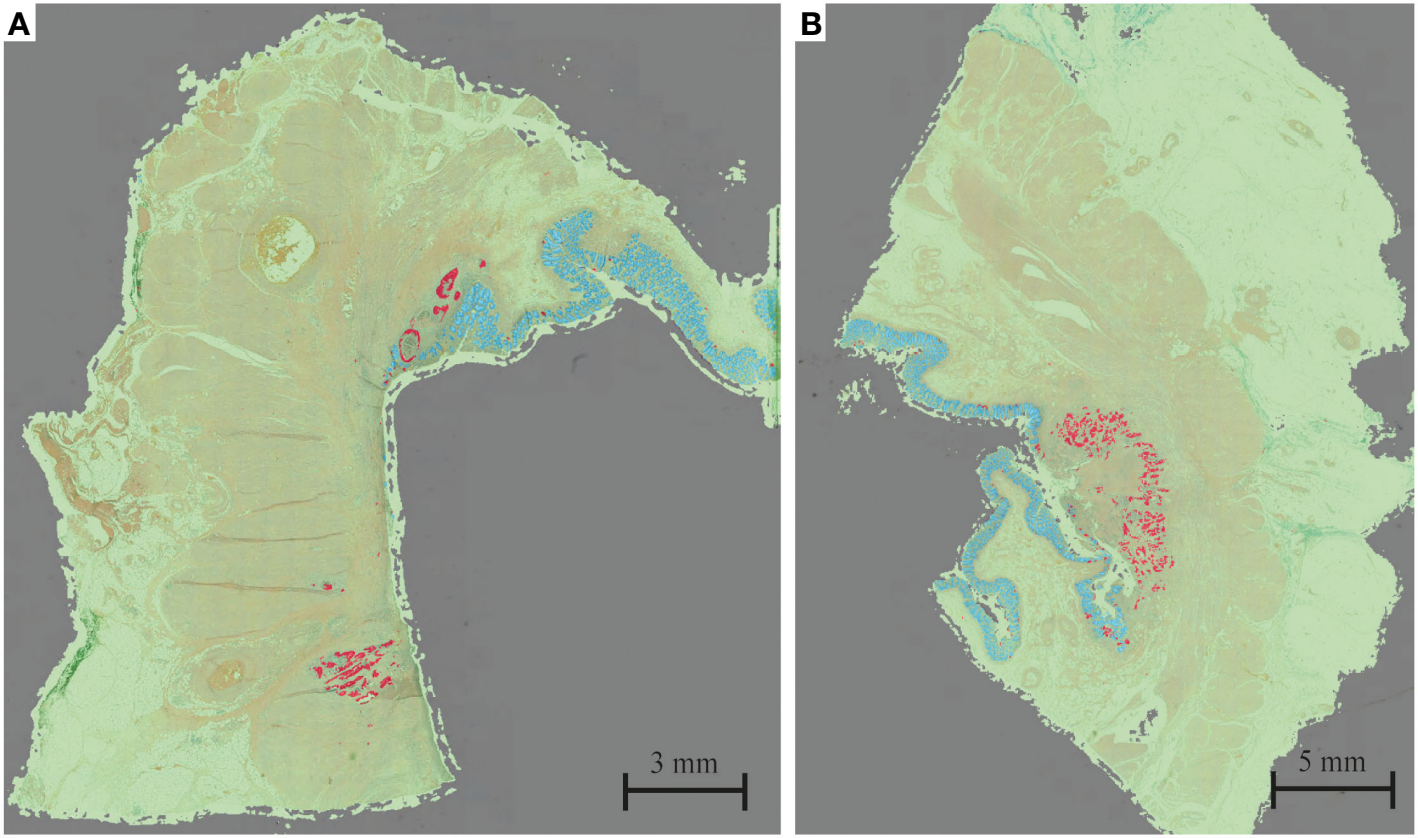
Example of **(A)** tumor fragmentation and **(B)** tumor shrinkage. Red = tumor; blue = mucosa; green = other tissue; gray = background.

The HALO tissue classification algorithm identified tumor (red), normal mucosa (blue), other tissue types (green) and background (grey) in each histological slide (**Figure 1**), which was used for a quantitative analysis of the tumor response pattern (**Table 2**). Examples of the two main tumor response patterns, tumor fragmentation and shrinkage are shown in **Figure 2**.

**Table 2:** Tumor response patterns based on quantitative analysis of histopathology slides. Data is presented as median with range (smallest to largest).

	Tumor regression grade 2	Tumor regression grade 3	p-value
Total, *n*	17	13	
Tumor response			
Fragmentation, *n*	11	5	
Shrinkage, *n*	6	8	
Fragmentation: maximum width of isolated fragments, *mm*	0.68 (0.06 – 6.90)	1.80 (0.28 – 5.60)	0.054
Shrinkage: maximum width of tumor bulk, *mm*	4.60 (0.18 – 10.90)	7.55 (1.70 – 14.90)	0.019
Shortest distance between vital tumor and luminal surface, *mm*	0.073 (0.001 – 3.30)	0.116 (0.002 – 8.90)	0.36
Area of vital tumor (1 central slide), *mm^2^*	1.92 (0.17 – 6.47)	4.81 (1.09 – 30.99)	0.005
Volume of vital tumor (all tumor containing slides), *mm^3^*	5.72 (0.93 – 25.80)	14.27 (1.87 – 74.87)	0.02

Overall, the response pattern was of shrinkage type in 14 patients and of fragmentation type in 16 patients. For both groups combined, the largest vital tumor fragment per patient was smaller than 1 mm for 38% of patients, below 0.2 mm for 12% of patients and for one patient was as small as 0.06 mm. For 29% of patients residual vital tumor was present within the first 0.01 mm from the luminal surface and for 87% within the first 0.5 mm. In one patient there was 8.9 mm of healthy tissue between the residual vital tumor tissue and luminal surface. Moreover, invasion depth for both Mandard TRG 2 and TRG 3 were similarly distributed.

### 3.2 Tumor fragmentation

**Figure 3:**
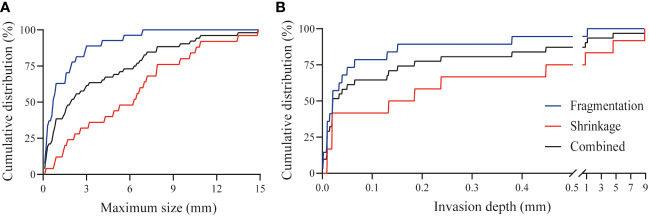
Tumor characteristics after CRT. **(A)** Cumulative distribution of the maximum width of tumor fragments in case of tumor fragmentation (n=16, blue line), tumor shrinkage (n=14, red line) and combined cohort (n=30, black line) after CRT. For both groups combined, the largest vital tumor fragment per patient was smaller than 1 mm for 38% of patients, below 0.2 mm for 12% of patients and for one patient was as small as 0.06 mm. **(B)** Cumulative distribution of the minimum invasion depth from the luminal surface in case of tumor fragmentation (n=16, blue line), tumor shrinkage (n=14, red line), and the combined cohort (n=30, black line) after CRT. For 29% of patients residual vital tumor was present within the first 0.01 mm from the luminal surface and for 87% within the first 0.5 mm. In one patient there was 8.9 mm of healthy tissue between the residual tumor tissue and the luminal surface.

The response pattern was of fragmentation type in 65% and 38% in TRG 2 and TRG 3 cases, respectively. The median size of the widest isolated tumor fragments per patient was 0.68 mm for TRG 2 cases, and 1.80 mm in TRG 3 cases. In 63% of patients the widest fragments measured below 1.0 mm and 78% below 2.0 mm in size (**Figure 3A**). Residual tumor fragments were widely spread throughout the original tumor bed, encapsulated by fibrotic tissue. Individual tumor fragments could be as small as 0.06 mm.

### 3.3 Tumor shrinkage

The response pattern was of shrinkage type in 35% and 62% in TRG 2 and TRG 3 cases, respectively. The median value of the tumor width per patient was 4.60 mm and 7.55 mm in TRG 2 and TRG 3 cases, respectively. The spread of the width was very large, varying from several hundred micrometers to 1.5 centimeters. Detailed analysis showed that the width of the residual vital tumor bulk was smaller than 1.0 mm in 12% of cases, whereas 50% was smaller than 6.3 mm (**Figure 3A**).

### 3.4 Tumor invasion

Residual vital tumor was observed in all layers of the intestine. The most common location was the submucosa or muscle layers, within 1 mm of the mucosal lining of the rectum (ypT1-2), however, vital tumor fragments were also observed in the mesorectal tissue, and extending into other organs (ypT3-4). The tumor invasion depth varied between several micrometers and up to 8.9 mm from the mucosa. In case of fragmentation, residual tumor fragments were present within 0.5 mm from the luminal surface in 95% of cases, whereas for shrinkage this was less, i.e. 75% (**Figure 3B**).

## 4 Discussion

This study provides an overview of the quantitative histopathological characteristics of the size and depth distributions of residual vital tumor tissue after CRT treatment in rectal cancer. In our study population, 63% of patients with a TRG 2 response after CRT harbor residual vital tumor fragments of less than 1 mm. Importantly, vital tumor fragments were mostly present within 0.5 mm of the luminal surface, yet could also be located in the mesorectal tissue and extending to other organs. However, in case of a TRG 2 response of large primary tumors, vital tumor fragments were observed extending up to 8.9 mm from the luminal surface.

For years, assessing treatment response in rectal cancer has been investigated intensively, focusing on clinicopathological characteristics or biomarkers as predictors, with mixed results (24, 25). For that reason, alternative approaches for response assessment, such as (novel) optical imaging techniques, should be explored. Optical imaging techniques use light to obtain highly detailed images and signals of organs, tissues, cells or molecules in a minimally invasive or non-invasive way. Optical imaging techniques harbor many advantages, such as the capability of high resolutions, high specificity for set targets and feasibility for real-time imaging. Moreover, optical imaging has the additional benefit that they lack harmful radiation and can therefore be used repeatedly for monitoring of disease progression or treatment effect.

**Figure 4:**
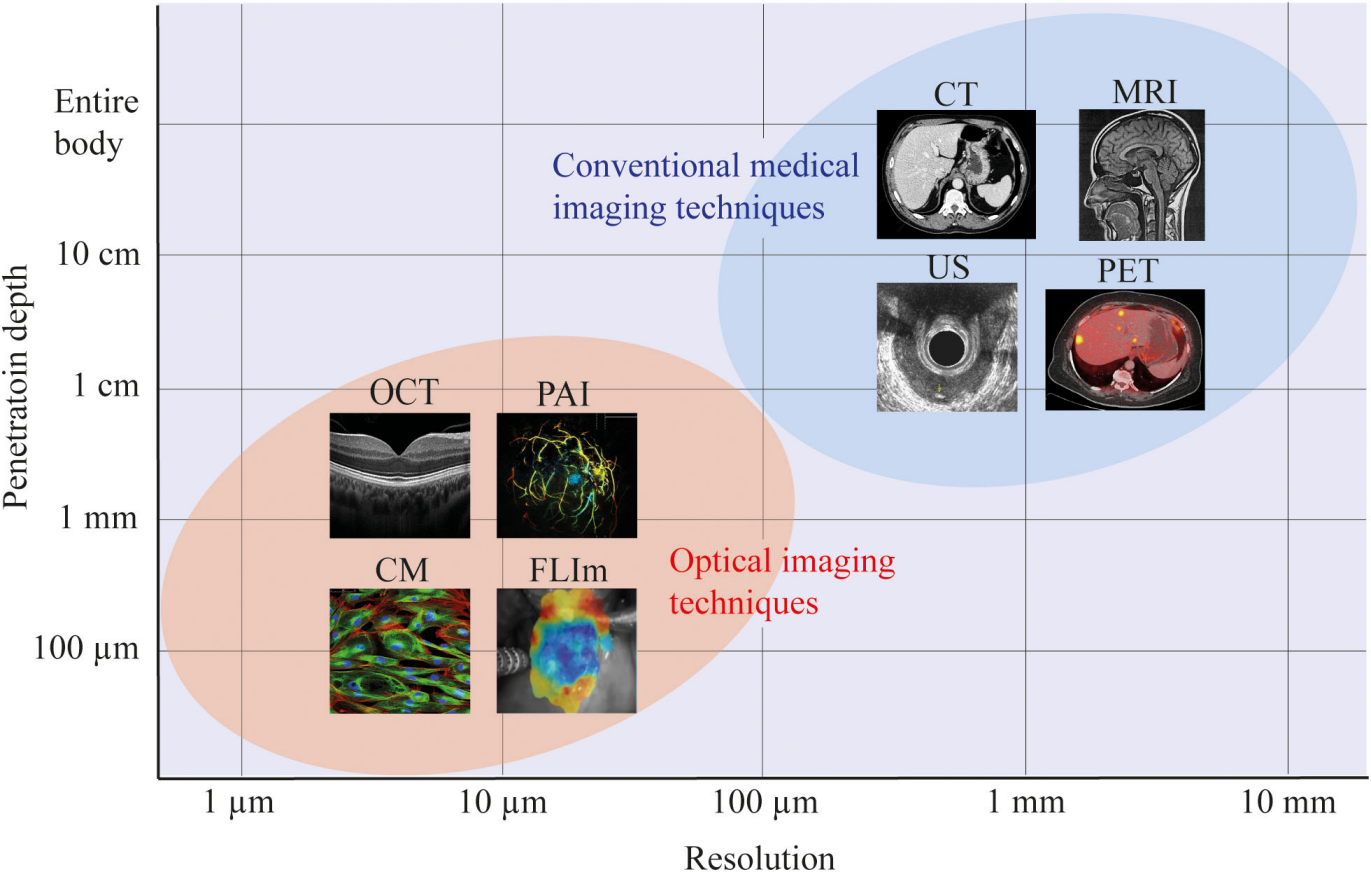
Resolution and penetration depths of several imaging modalities. For most optical imaging modalities there is a trade-off between the resolution and imaging depth, achieving more accurate resolutions, at the cost of penetration depth within the tissue of interest. In this figure, a schematic representation is used to indicate the approximate resolution and penetration depth of optical imaging versus conventional medical imaging modalities. For all imaging modalities, the exact resolution and penetration depth will depend on the specific setup. CM, confocal microscopy; FLIm, fluorescence lifetime imaging; OCT, optical coherence tomography; PAI, photoacoustic imaging; US, ultrasound; CT, computed tomography; MRI, magnetic resonance imaging; PET, positron emission tomography.

As illustrated in **Figure 4**, the demand for high resolutions (<1 mm) required to detect small tumor fragments can be achieved with optical imaging. **Figure 4** presents only a small sample of available optical imaging techniques, each with their own specific biochemical or structural targets. For example, optical coherence tomography (OCT) uses the refractive properties of light waves in tissue to provide visualization of cross-sectional and 3D images of tissues (26), fluorescence lifetime imaging (FLIm) provides information about the biochemical composition of tissues by measuring the decay of fluorescent molecules (27) and photoacoustic imaging (PAI) utilizes laser-generated ultrasound waves to display tissue morphology and vasculature (28).

It is important to realize that there is an inherent trade-off between resolution and imaging depth. By selecting a technique with a higher resolution, the imaging depth will decrease. However, most optical imaging setups allow for interchanging these parameters, thereby selecting the desired resolution and imaging depths for a specific application. The results from our analysis offers the theoretical framework to evaluate the prospects of different optical imaging techniques. Moreover, challenges such as limitation in field of view, resolution (29) or feasibility for *in vivo* use (30, 31) have been addressed by multiple studies, providing solutions and opportunities for further research. For example, techniques as hyperspectral laparoscopes (32), confocal laser endomicroscopy (33) and tethered capsules (34) have indicated the potential of optical imaging in endoscopic use. Despite these advances, a commercially available optical imaging device is not yet available for tumor response assessment in rectal cancer. Hence, exploration of optical imaging techniques could be the way forward towards accurately defining treatment response assessment in rectal cancer patients. However, it is important to realize that implementation of any technique in the rectal cavity needs an optimized design for intended use.

While the results presented in this study show the potential for optical imaging in treatment response assessment, they also explain why it is difficult to discriminate between a good clinical and a complete response using conventional clinical examination. The foremost reason is that CRT can result in submillimeter residual tumor fragments below the resolution of MRI, and can be scattered throughout the intestinal tissue layers, rendering them invisible for endoscopy.

Currently, MRI is the golden standard for treatment response assessment in rectal cancer. While conventional MRI can achieve a resolution of approximately 1 mm (**Figure 4**), this is insufficient to identify the submillimeter tumor fragments demonstrated in the present study. Moreover, CRT-induced fibrosis in the tumor bed, replacing vital tumor, decreases the accuracy of MRI to detect residual viable tumor due to the lack of contrast between fibrosis and tumor fragments (35–37). As such, the dimension and distribution of tumor fragments, together with the surrounding fibrosis provides a big challenge for radiologists to accurately assess treatment response with MRI. Currently it is possible to improve this resolution using Ultra High Field (UHF) 7-9 Tesla machines, achieving a resolution below 0.5 mm (38). Even so, in 21% of our patients the largest tumor fragments was less than 0.5 mm. Furthermore, the problem of distinguishing fibrosis from tumor in these UHF scans remains. Another possibility for improving the diagnostic capability of MRI for response imaging of rectal cancer is the use of diffusion-weighted imaging (DWI). DWI offers the possibility to visualize a functional parameter utilizing the diffusion of water molecules within tissues. The advantage of DWI would be the possibility to increase the contrast between tumor and fibrosis. In assessment of breast and renal cancer response to CRT, DWI improved the evaluation of treatment response (39–41). However, the limiting factor of the resolution with respect to the small size of the fragments remains.

In addition to shrinkage and fragmentation of the tumor, in approximately 20% of patients undergoing CRT, microscopic intramural spread (MIS) is present (42), i.e. residual tumor extension beneath normal appearing mucosa. In clinical practice, MIS is commonly used for planning additional radiotherapy, or take into account when selecting the resection plane around the visible tumor. Whilst multiple studies have focused on retrospective assessing the MIS after CRT (42–44), intraoperative assessment remains challenging due to the limited size of the residual tumor fragments in most cases. Moreover, a tumor-positive circumferential resection margin (CRM) after CRT remains an important prognostic factor for local recurrence and overall survival, and can be as high as 31.8% (45). Hence, intraoperative assessment of the MIS potentially allows for more accurate selection of the resection plane, and (novel) optical imaging techniques could provide the tools to decrease tumor-positive CRM rates.

In this study, an AI algorithm was trained for labelling of residual tumor in pathology slides of rectal cancer. Such an algorithm enables the analysis of a large number of slides in detail. Moreover, the same analysis by a pathologist would have taken up a considerable amount of time. In future studies with (novel) imaging techniques, such an AI algorithm could provide key insights into the performance of these techniques by providing detailed histopathological information of the imaged tissue. Furthermore, our detailed analysis revealed that invasion depth of residual tumor fragments/bulk was not related to the Mandard TRG. However, most patients with a TRG 3 response displayed larger tumor volumes and larger tumor diameter than TRG 2 tumors. Thus, Mandard TRG is not only a measure of response, but can also be a measure of residual tumor burden.

There are some limitations to this study. First, 30 patients were included, resulting in 198 pathology slides. The novelty of the segmentation tool required us to manually check the performance of the segmentation in every segmented slice, resulting in a lower patient population. Secondly, an uncertainty remains about what portion of the residual tumor in the histological assessment is still vital and can result in a regrowth. However, in this study we assumed that our assessment of the vital portion of the tumor is right. Moreover, it is important to keep in mind that the AI algorithm was only trained on 3 completely annotated slides, while many more are needed optimizing the algorithm for general application. This could be seen as a limitation, since the trained AI for this study might lack robustness for independent analysis of histology slides. However, the AI was used as a tool for manual annotation, and not as a replacement for an experienced pathologist, and therefore the minimal training size was adequate for the application as used here. Furthermore, the quantitative measurements reported in this paper were verified manually to be accurate, and thus do not rely heavily on the AI for segmentation borders. Moreover, the AI performed well and no major adjustment had to be done to the segmentation once trained.

For successful organ-sparing treatment, it is crucial that clinicians can accurately identify the optimal treatment for each patient based on the response of the tumor to CRT. Resolutions of several micrometers are required for visualizing residual tumor fragments, and an imaging depth of several millimeters is essential for detecting fragments in all layers of the rectal wall. Moreover, it is important to realize that the histopathological characteristics of the tissue are paramount in the selection of an imaging technique. For *in vivo* application, it is important to realize that the imaging technique has to be implemented in an endorectal probe for optimal access to the tumor (46). Many optical imaging techniques have been transformed from table-top setups to endoscopic imaging probes, showing the potential of optical imaging techniques for colorectal response assessment (33, 34, 47–49). results presented in this study show that conventional imaging methods (mainly CT and MRI) lack the resolution for detecting residual vital tumor after CRT in rectal cancer, and hence have limited value in the therapeutic decision-making process around W&W in clinical practice. Our results, however, provide a theoretical basis for novel research in imaging techniques that can achieve the needed resolutions. Depending on the exact application, optical techniques have their own benefits over conventional CT and MR imaging.

To summarize, optical imaging techniques have the prospect of becoming a complimentary tool next to conventional methods for rectal cancer response assessment, since these techniques offer high-resolution imaging with enhanced contrast between tissue types.

## Data availability statement

The raw data supporting the conclusions of this article will be made available by the authors, without undue reservation.

## Ethics statement

The studies involving humans were approved by Institutional Review Board of the Netherlands Cancer Institute. The studies were conducted in accordance with the local legislation and institutional requirements. The ethics committee/institutional review board waived the requirement of written informed consent for participation from the participants or the participants’ legal guardians/next of kin because written informed consent was not required according IRB guidelines.

## Author contributions

Study design: AP and TR. Data collection: SS, JB, PS, IS, BG, GB, and AP. Data interpretation: SS, JB, PS and AP. Writing manuscript: SS, AP, and MW. Reviewing manuscript: JB, PS, IS, BG, GB and TR. All authors read and approved the final manuscript.
